# E-Learning and North-South collaboration: the experience of two public health schools in France and Benin

**Published:** 2009-10-14

**Authors:** Guévart Edouard, Billot Dominique, Paraïso Noël Moussiliou, Guillemin Francis, Bessaoud Khaled, Briançon Serge

**Affiliations:** 1 Service de Coopération et d’Action Culturelle, French Embassy, Cotonou, Bénin;; 2 Institut Régional de Santé Publique (IRSP), Ouidah, Bénin;; 3 Ecole de Santé Publique de Nancy, Faculté de Médecine, Vandoeuvre les Nancy, France.

**Keywords:** developing countries, distance learning, e-learning, North-South collaboration, partnership, public health

## Abstract

**Introduction:**

Distance learning (e-learning) can facilitate access to training. Yet few public health E-learning experiments have been reported; institutes in developing countries experience difficulties in establishing on-line curricula, while developed countries struggle with adapting existing curricula to realities on the ground. In 2005, two schools of public health, one in France and one in Benin, began collaborating through contact sessions organised for Nancy University distance-learning students. This experience gave rise to a partnership aimed at developing training materials for e-Learning for African students. The distance-learning public health course at Nancy teaches public health professionals through a module entitled “Health and Development.” The module is specifically tailored for professionals from developing countries. To promote student-teacher exchanges, clarify content and supervise dissertations, contact sessions are organized in centres proximate and accessible to African students. The Benin Institute’s main feature is residential team learning; distance-learning courses are currently being prepared.

**Outcome:**

The two collaborating institutions have developed a joint distance-learning module geared toward developing countries. The collaboration provides for the development, diffusion, and joint delivery of teaching modules featuring issues that are familiar to African staff, gives the French Institute credibility in assessing research work produced, and enables modules on specific African issues and approaches to be put online.

**Lessons learned:**

While E-learning is a viable educational option for public health professionals, periodic contact can be advantageous. Our analysis showed that the benefit of the collaboration between the two institutions is mutual; the French Institute extends its geographical, cultural and contextual reach and expands its pool of teaching staff. The Benin Institute benefits from the technical partnership and expertise, which allow it to offer distance learning for Africa-specific contexts and applications.

## Introduction

The death of health resources in the developing world is particularly severe in sub-Saharan Africa. The World Health Organization (WHO) 2006 report devoted to this issue emphasises the chronic shortage of qualified healthcare workers [1]. The public health sector is likewise concerned and its teaching institutions are unable to meet needs for qualified professionals. The WHO report advocates distance learning to help close this gap by facilitating access to training.

Distance learning affords geographically isolated professionals opportunities to develop new skills while on the job [2] gives participants freedom in organising their training while requiring considerable autonomy, time-management, documentation, and self-assessment [3]. To avoid isolation, numerous authors highlight the importance of including social links: collaborative assignments, tutoring, contact sessions [4, 5].

In the public health sector, training institutions in developing countries experience difficulties when trying to establish on-line curricula, while developed ones struggle to adapt existing curricula to realities on the ground with which they are not familiar [6]: thus, partnerships to develop tailored training courses in developing countries.

A course conducted by the school of public health of Nancy University in France (Ecole de Santé Publique, ESP) and the Regional Institute for Public Health in Ouidah, Benin (Institut Régional de Santé Publique, IRSP) is a case study. In 2005, these partner institutions, both involved in training public health officers for Africa, began collaborating with contact sessions organised for Nancy University distance-learning students in public health. We aim to show how collaboration promotes the development of a mutually beneficial partnership, widening and consolidating the training offerings for Africa.

## Context and methods

The distance-learning public health diploma in ESP (Diplôme Universitaire de Santé Publique et Promotion de la Santé, DUSPPS) created in 1987 was followed by an E-learning version in 2004 [7]. The objectives of these programs are to help healthcare and social professionals acquire conceptual and methodological skills in public health, including the design, implementation, development, and evaluation of programmes at different levels. A module entitled “Health and Development” now targets professionals from developing countries. The curriculum mainly covers cognitive learning principles and context-related problem-solving using online tools, with case studies explored individually or in virtual groups. Three contact sessions, one of which is compulsory, over a 2-year period promote exchanges among students and teachers, clarify online contents, and facilitate the supervision of the drafting of dissertations. The examination occurs during the compulsory session. Sessions can be in Nancy or in African collaborating centres. The Nancy School is developing active partnerships with public health institutions to provide greater access to African students.

Team learning has been central to the teaching policy since the creation of IRSP for African public health professionals in Benin in 1977. The training is a 12-month residential course with field activities. Only one short course (vaccinology), is offered by IRSP through distance-learning; the rest, including a 4-semester Master’s degree, are planned for introduction.

IRSP-ESP distance-learning contact sessions run for one week. The training contents and methods are evaluated by students (satisfaction questionnaires and nominal group), and the two institution teams use the sessions to assess activities and plan future collaborations. The results presented here are derived from student evaluations and other documents from both institutions.

## Outcome

### Contact sessions

Of the 149 students enrolled in 2006–2007, 36 applied for the Ouidah session in 2007 and 28 attended. The students came from Benin, Burkina Faso, Cameroon, the Democratic Republic of Congo, Côte d’Ivoire, Djibouti, Gabon, Ghana, Mali, Niger, Rwanda, and Senegal. Staff members at the two schools conducted the session jointly. Two days were devoted to clarification of curriculum content and joint correction of a previously assigned exercise. The main clarifications requested by students focused on health education/prevention, statistics and standardisation methods, definition of objectives for investigation, action and evaluation. The examination included the critical analysis of an article; A two-day workshop aimed to identify and analyse the problem for the dissertation. Finally there was a lecture-debate on distance-learning, also involving partner institutions.

### Evaluation by Students

Among session features, students found the following worthwhile: communication and psycho-social components of encounters with other students and staff, group assignments, interactive methods, cognitive content and its clarification, and novel modes of functioning. Students reported that they would have liked a longer contact session for further revision, more exercises to prepare for the examination, longer examination time, and a second compulsory session involving their virtual group tutors. They were unanimous on the value of the sessions. The negative points concerned certain material or logistical aspects.

### Pedagogical collaboration

Meetings involving the two ESP teachers and the IRSP team showed their agreement on teaching methods and approaches to public health. A future collaboration was planned.

IRSP appointed two teachers to tutor the diploma and supervise/assess ESP dissertations. The institutions agreed on joint coordination of the distance-learning Master’s degree according to the West African Health Organization recommendations for the university reform: joint development of modules was planned targeting central themes for developing countries, including reproductive health, managing epidemics, pharmaceutical policies, and catering to displaced populations. It was agreed that regular exchanges via joint tutoring and pooling of modules would be conducted for the oversight of all aspects of the curricula.

## Lessons learned

To date, few E-learning experiments in public health have been conducted. [2, 7] and a dearth of health professionals remains a problem in many parts of the world, particularly in Africa. However, despite being a priority for the continent [8], training large numbers of professionals cannot be easily accomplished using the classical residential model [9]. Rather, it is recommended that African institutions develop academically rigorous, internationally validated courses via inter-institutional collaborations. This will allow for the integration of large number of people into a professional network. For this purpose distance-learning is unanimously advocated [1].

There are several limitations to distance-learning. Perhaps most challenging is the coordination of training with professional and personal agendas. Isolation is also a significant challenge students report feeling disconnected from the training content and complain that they cannot compare their skills with others [10]. Short contact sessions can help promote the socio-affective and socio-cognitive dimensions of learning, fostering a feeling of belonging [11]. This aspect emerged clearly in student evaluations in Ouidah, Benin. By reducing relational distance, encounters facilitate working relationships and help express the tacit knowledge present in professional practice, which is easier to grasp in direct exchanges. By working closely, individuals understand one another and establish a dialogue where non-verbal communication helps reveal diversity in reasoning and approach [3, 12]. Pooling knowledge and practices is fostered by actual contacts, which constitute lessons in themselves [13, 14]. Furthermore, some course content is difficult to implement via distance-learning, such as the interaction required in defining the research theme.

Present trends favour mixed systems combining distance-learning with the positive contributions of tutoring and contact sessions [2]. Contact sessions in a distance-learning system should focus on appropriation of the course system, links with the institution and the supervising team, and encounters among students [4, 15].

For ESP African students the most obvious advantage of contact organized in collaborating centers is the reduction of costs and administrative procedures for African nationals due to the proximity of the centers.

The specific field knowledge of the Benin staff should also be underlined. In keeping with international priorities, the IRSP/ESP agreement provides for the development, diffusion, and joint delivery of teaching modules relating to major themes for Africa, including: infectious diseases, mother and child survival, HIV-AIDS epidemic, access to medication, access to care, and care quality. These themes, familiar to the Benin staff, will give the Nancy “Health and Development” module greater credibility for the assessment of research work produced [6].

ESP has 20 years’ experience in distance-learning and several in E-learning: distance-learning collaboration between IRSP and its Northern partners will enable IRSP modules on specific African issues and approaches to be put online [7, 16]. Thus, the IRSP/ESP partnership extends its common pool of pedagogical and technical skills applied to specific public health issues in Africa. Nevertheless, this type of course in Africa can encounter severe constraints limiting impact or biasing analysis.

Accessing course content can be problematic for students in deprived areas where electricity, Internet access, and time may be in short supply. These difficulties are compounded in interactive online systems where line stability is essential. Teaching institutions should exercise leniency with students, while still adhering to academic rules and deadlines. Technical barriers also exert severe selective pressure on students to sustain motivation despite practical difficulties and the resulting isolation [17–19].

Travel to contact sessions can be difficult, even within Africa. Flying can be cumbersome and costly, and administrative formalities can be complicated for some. Thus students actually able to attend were probably not fully representative, which biases the favourable assessments noted.

There are also inequalities between the two institutions. These concern access to documentation, modes of staff recruitment, resources, salaries, and methods. The mutually beneficial partnership could suffer from these differences unless staffs concerned have strong personal incentive.

## Conclusion

The provisional conclusions of this experience show that despite significant limitations E-learning is a viable training tool for public health professionals and that periodic contact is advantageous for both institutions. ESP gains from geographical, cultural, and contextual accessibility and extends its pool of teaching staff. IRSP gains from the technical partnership and expertise in E-learning, thereby enabling it to offer distance-learning for Africa-specific contents and applications. They can now move on to an online Master’s degree curriculum comprised of shared modules. They can also capitalize on student experiences via tutorials, providing a wider scope for case studies and research.

Training institutions could be recommended to put together infrastructures, experience, and pedagogical failures, to combine distance-learning with contact sessions in order to offer their students individually tailored courses via modules developed jointly and geared toward issues that professionals actually encounter.
